# Development of Highly Luminescent Water-Insoluble Carbon Dots by Using Calix[4]pyrrole as the Carbon Precursor and Their Potential Application in Organic Solar Cells

**DOI:** 10.1021/acsomega.2c01795

**Published:** 2022-05-24

**Authors:** Yağız Coşkun, Fatma Yelda Ünlü, Tuğbahan Yılmaz, Yurdanur Türker, Abdullah Aydogan, Mahmut Kuş, Caner Ünlü

**Affiliations:** † Department of Nanoscience and Nanoengineering, Istanbul Technical University, Maslak, 34469 Istanbul, Turkey; ‡ Faculty of Science and Letters, Department of Chemistry, Istanbul Technical University, Maslak, 34469 Istanbul, Turkey; § Vocational School of Technical Sciences, Department of Electricity and Energy, Konya Technical University, Selcuklu, 42150 Konya, Turkey; ∥ Sabanci University Nanotechnology Research & Application Center (SUNUM), Sabanci University, Istanbul 34956, Turkey; ⊥ Department of Chemical Engineering, Konya Technical University, 42075 Konya, Turkey; # Istanbul Technical University Nanotechnology Research and Application Center (ITUNano), Istanbul 34469, Turkey

## Abstract

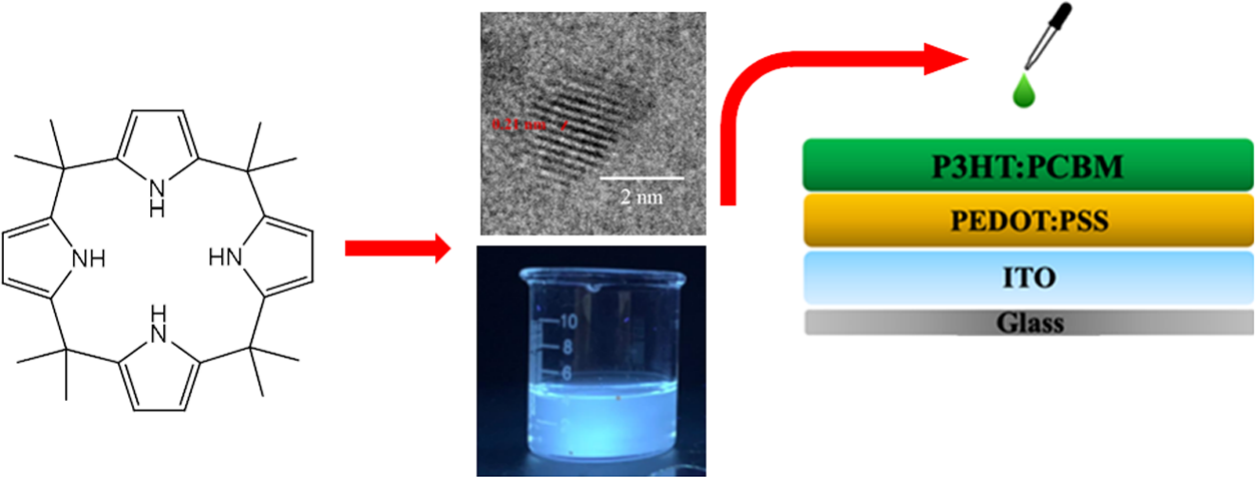

Carbon dots (CDs) are carbon-based fluorescent nanomaterials that are of interest in different research areas due to their low cost production and low toxicity. Considering their unique photophysical properties, hydrophobic/amphiphilic CDs are powerful alternatives to metal-based quantum dots in LED and photovoltaic cell designs. On the other hand, CDs possess a considerably high amount of surface defects that give rise to two significant drawbacks: (1) causing decrease in quantum yield (QY), a crucial drawback that limits their utilization in LEDs, and (2) affecting the efficiency of charge transfer, a significant factor that limits the use of CDs in photovoltaic cells. In this study, we synthesized highly luminescent, water-insoluble, slightly amphiphilic CDs by using a macrocyclic compound, calix[4]pyrrole, for the first time in the literature. Calix[4]pyrrole-derived CDs (CP-DOTs) were highly luminescent with a QY of over 60% and size of around 4–10 nm with graphitic structure. The high quantum yield of CP-DOTs indicated that they had less amount of surface defects. Furthermore, CP-DOTs were used as an additive in the active layer of organic solar cells (OSC). The photovoltaic parameters of OSCs improved upon addition of CDs. Our results indicated that calix[4]pyrrole is an excellent carbon precursor to synthesize highly luminescent and water-insoluble carbon dots, and CDs derived from calix[4]pyrrole are excellent candidates to improve optoelectronic devices.

## Introduction

Carbon dots (CDs) are versatile nanomaterials with excellent properties such as easy and cost-effective synthesis, high fluorescence quantum yield, and, more importantly, less toxicity compared to their heavy metal-based alternatives.^1−5^ Due to their low toxicity, CDs are mostly used in biotechnology and thus synthesized in hydrophilic form.^1−8^ However, hydrophilic CDs possess a high amount of surface defects on the nanocrystal surface compared to their metal-based alternatives, and as a consequence, they have much more defect energy states, which causes a decrease in the quantum yield of CDs and also disrupts the charge transfer in photovoltaic cells.^1−5,9−11^ Therefore, preparation of hydrophobic/amphiphilic CDs with high quantum yields to be used in photovoltaic applications, such as organic solar cells, has become an important challenge.^12−15^

In early studies, amphiphilic carbon dots were synthesized by using noncommercial carboxylic acid derivatives having long hydrocarbon tails, which can be prepared in multiple synthesis steps.^16^ Later, Zhu et al. developed an easier hydrothermal bottom-up method to synthesize amphiphilic carbon dots by using xylose and ethylenediamine as carbon precursors and *N*,*N*-dimethylacetamide as the solvent.^17^ Also, amphiphilic CDs were synthesized by using several organic solvents (like toluene, dimethylformamide, acetonitrile, etc.), which act as the carbon precursor and solvent together, through hydrothermal synthesis methods.^18,19^ However, the highest fluorescence quantum yield, an important indicator to determine the quality of carbon dots, obtained in these studies was around 35%,^16−19^ which was not comparable to the quantum yields of metal-based quantum dots.^18,19^ Different single or multiple carbon precursor systems, such as citric acid + phenylenediamine, cetylpyridinium chloride, cetylpyridinium bromide, and octadecylamine + glucose, were used to synthesize amphiphilic or hydrophobic CDs with high fluorescence quantum yield,^20−25^ but the highest obtained quantum yield was around 47%,^25^ which can be still considered low compared to the quantum yield of their metal-based alternatives.^18,19^

In this study, we synthesized highly luminescent (quantum yield > 60%), water-insoluble, slightly amphiphilic carbon dots (CP-DOTs) with graphitic structure and size around 4–10 nm by a modified hydrothermal synthesis method using octamethylcalix[4]pyrrole (CP, C_28_H_36_N_4_, will be referred as calix[4]pyrrole or CP in further text) as the carbon precursor and toluene as the solvent. CP-DOTs had significantly higher quantum yields compared to different amphiphilic/hydrophobic CDs obtained by using either different carbon precursors or different solvents. CP-DOTs were also soluble in various polar and nonpolar organic solvents (e.g., toluene, n-hexane, methanol, and ethanol), showing their amphiphilic characteristics. In brief, our method proposed utilization of a valuable supramolecule, calix[4]pyrrole, as the carbon precursor in a standard hydrothermal carbon dot synthesis procedure and development of water-insoluble carbon dots with very high quantum yield (above 60%) for the first time in literature.

Then, CP-DOTs were used as photoactive layer additives in various concentrations to improve the electrical parameters of organic solar cells (OSCs). CP-DOTs were introduced to the photoactive layer, poly(3-hexylthiophene-2,5-diyl) (P3HT):(6, 6)-phenyl C61 butyric acid methyl ester (PCBM) blend, to improve the charge transport properties and absorption ability.^26−30^ The addition of CP-DOTs enhanced the morphology and crystal structure of the photoactive layer, which led to an improved power conversion efficiency (PCE) compared to the reference device (nondoped P3HT:PCBM). The highest PCE of 3.39% was achieved with a fill factor of 51.15%, *a*_oc_ of 0.571 V, and *J*_sc_ of 9.113 mA cm^–2^ after addition of a 3 vol % CP-DOT additive. These results showed that introducing CP-DOTs into the photoactive layer significantly improves the OSC parameters.

## Materials and Methods

### Synthesis of Carbon Dots

#### Synthesis of Calix[4]pyrrole-Based Carbon Dots

In this study, carbon dots were synthesized through a modified hydrothermal synthesis method. The main carbon precursor, CP, was synthesized as described in the literature before.^31^ All of the chemicals were purchased from Sigma-Aldrich with highest purity unless otherwise mentioned.

Carbon dots were prepared as follows; 20 mg of CP was dissolved in 20 mL of toluene in a 50 mL beaker at 60 °C. Afterwards, the solution was transferred to a Teflon-lined autoclave, sealed, and kept in a furnace oven at 200 °C for 8 h. Then, the autoclave was taken out from the furnace and cooled down to room temperature to stop the nanocrystal growth. A yellow crude solution was obtained at the end of the reaction. To purify the carbon dots, the crude solution was filtered through a filter paper with 10 μm pores and then filtered through a filter syringe with 0.22 μm pores. Each filtration step was repeated twice. In the final step, carbon dots were centrifuged at 14 000 rpm for 30 min to get rid of the remaining big clusters and aggregates in the solution. The supernatant was then taken into a beaker, the purified carbon dot solution was dried, and the solid product was kept in a refrigerator at 2 °C. The calix[4]pyrrole-based carbon dots will be referred as CP-DOTs in further discussion. The same procedure was repeated to synthesize calix[4]pyrrole-based carbon dots by using hexane as the solvent instead of toluene, and the resulting product was named as H:CP-DOT.

#### Synthesis of Carbon Dots by Using Toluene or Pyrrole as the Carbon Precursor

The same procedure above was repeated to synthesize (1) carbon dots by using toluene as both the carbon precursor and solvent (T-DOTs), and (2) carbon dots by using pyrrole as the carbon precursor and toluene as the solvent (P-DOTs). The same experimental and purification processes were applied to each synthesis only by changing the carbon precursor, and the final products were kept in a refrigerator at 2 °C.

After the synthesis and purification of the product, the emission color of the carbon dots was observed under a UV lamp with an illumination wavelength of 366 nm. To characterize the optical properties, each carbon dot type was dissolved in an appropriate solvent and the absorption spectrum was recorded using a Scinco Neosys-2000 single-beam ultraviolet–visible (UV–vis) spectrophotometer. The excitation and emission spectra of each sample were recorded with a Varian Cary Eclipse Fluorescence Spectrofluorometer to elucidate the emission properties of each CD. The optical density of each carbon dot type was adjusted to 0.1 at 350 nm before collection of the emission and excitation spectra to avoid self-absorption. The quantum yield of each carbon dot was calculated by using Coumarin 102 as reference, as was described in the literature.^32−34^

High-resolution transmission electron microscopy (HR-TEM), X-ray photoelectron spectroscopy (XPS), and Fourier transform infrared spectroscopy (FTIR) measurements were conducted for the structural characterization of CDs. JEOL JEM-ARM200CFEG UHR-TEM was used to determine the size and crystal structure of CDs. XPS measurements were performed with Thermo Scientific K-Alpha with a monochromatic 1486.68 eV Al Kα X-ray line source and a 400 μm beam size to determine the elemental composition and bonding characterization of carbon dots. The surface characteristics of the carbon dots were determined by a PerkinElmer ATR-FTIR spectrophotometer.

### Fabrication of OSCs

**Figure 1:**
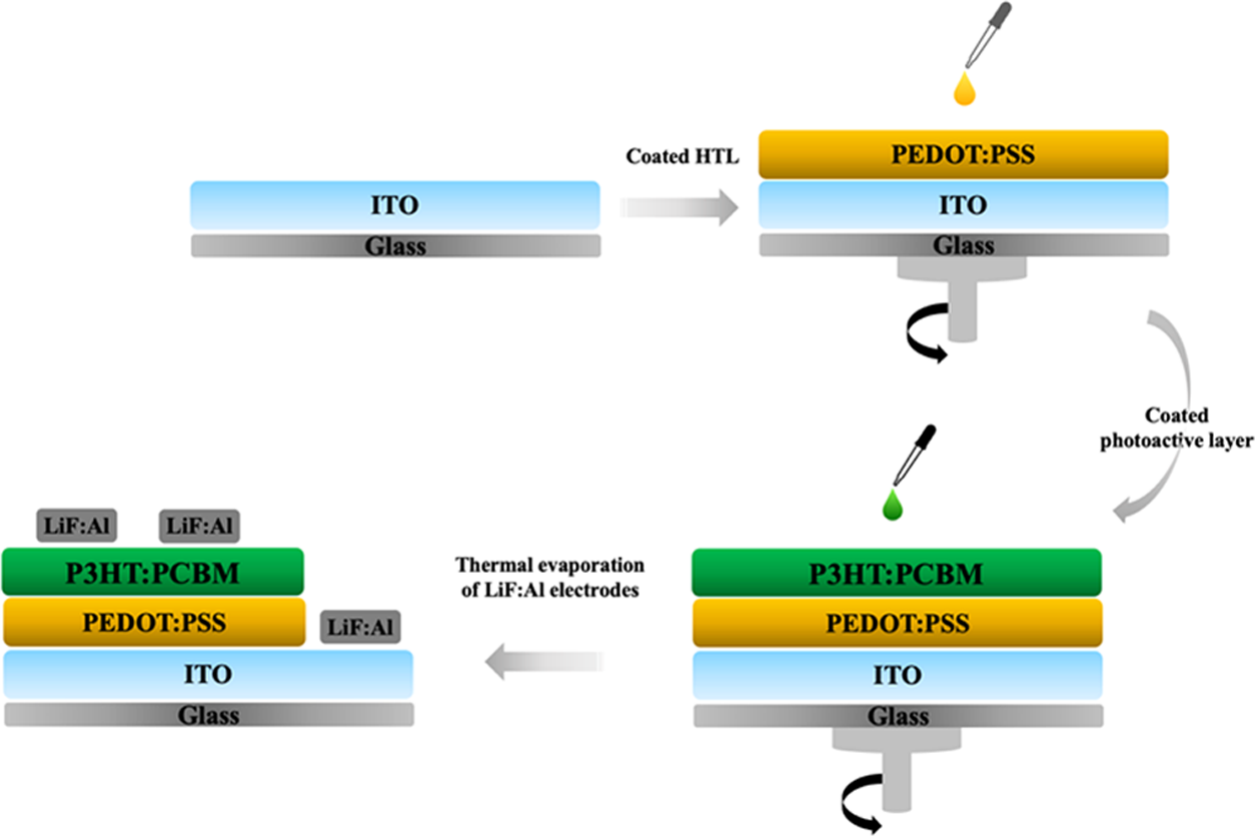
Schematic of the OSC fabrication.

The OSC devices were fabricated on indium-tin oxide (ITO)-coated substrates (1.5 cm × 1.5 cm) (Figure 1). Before constructing the organic solar cell device, all substrates were cleaned in a diluted Hellmanex detergent solution (Sigma-Aldrich, Hellmanex III), deionized water (DI), acetone, and isopropanol in an ultrasonic bath for 10 min. After drying with N_2_, all cleaned substrates were oxygen plasma treated in a Diener Plasma System Femto PCCE-Plasma Cleaner for 5 min to remove the residues.

All cleaned substrates were first spun at 3000 rpm for 20 s, and then spin-coated with poly(3,4-ethylenedioxythiophene):polystyrene sulfonate (PEDOT:PSS) at 2500 rpm for 20 s to form a hole transport layer (HTL). PEDOT:PSS-coated substrates were heated at 110 °C for 10 min. To form the photoactive layer, firstly P3HT (Sigma-Aldrich):PCBM (Lumtec) blend solution (weight ratio 1.0:0.6) was dissolved in chloroform (CF):chlorobenzene (CB) solution (1:1) at a concentration of 40 mg/mL. Afterwards, the P3HT:PCBM blend solution was stirred overnight at 70 °C. For the preparation of stock dopant solution, the CP-DOT solution was prepared by dissolving the CP-DOTs in toluene at a concentration of 10 mg/mL. Subsequently, different concentrations of CP-DOT solution were added into the P3HT:PCBM blend solution with different volume percents (1, 3, 5, and 7%) after filtration of the blend solution with a 0.22 μm poly(tetrafluoroethylene) (PTFE) filter. The CP-DOTs were completely dissolved in the prepared solutions without any trace of precipitation. All prepared P3HT:PCBM blend solutions, nondoped and doped, were spin-coated on the HTL layer at a speed of 2200 rpm followed by 2500 rpm for 30 s to form the photoactive layer and then baked on a hot plate at 110 °C for 20 min. Finally, 2 nm LiF and 80 nm Al electrodes were evaporated through a shadow mask (0.023 cm^2^ active area) under 2 × 10^–6^ mbar vacuum pressure to complete the OSC fabrication.

### Characterization of OSCs

The optical properties of P3HT:PCBM films were analyzed by an ultraviolet–visible (UV–vis) absorption spectrometer (Biochrom Libra S22) from 300 to 800 nm. To investigate the morphology of the P3HT:PCBM films nondoped or doped with CP-DOTs, an atomic force microscope (AFM, NT-MDT NTEGRA Solaris) was used in “tapping mode”. In order to understand the effect of the addition of CP-DOTs on P3HT:PCBM film crystallization, all P3HT:PCBM layers with nondoped and doped CP-DOTs were analyzed by an X-ray diffraction spectrometer (XRD, Bruker Advance D8) with Cu kα radiation at a wavelength of 1.5406 Å at 40 kV. The current density*–*voltage (*J–V*) curves of the fabricated OSCs were measured by an ATLAS solar simulator using a Keithley 2400 Source under the illumination of a simulated sunlight (AM 1.5, 80 mW cm^–2^) in the glovebox.

## Results and Discussion

### Synthesis of Water-Insoluble CDs

In general, amphiphilic/hydrophobic CDs can form in either crystalline or amorphous form.^25^ Depending on the structure, CDs would display different optical and electrical properties.^35^ Recently, it was shown that graphitic CDs transfer energy much more efficiently, and therefore it was concluded that graphitic CDs are much more effective constituents in photovoltaic cells and LEDs than their amorphous congeners.^35^ Amphiphilic/hydrophobic CDs may grow in either crystalline or amorphous form based on the structure of the carbon precursor. As the amount of sp^3^ hybridized carbon increases in the precursor, the CDs appear in amorphous form. However, as the sp^2^ (C)/sp^3^ (C) ratio in the precursor increases, the CDs appear in crystalline form.^25^ At this junction, we envisioned that calix[4]pyrrole, a commercially available macrocycle having four pyrrole rings connected to each other via methylene bridges, which has 16 sp2-hybridized carbon atoms in its pyrrole units, may be an excellent candidate as a carbon precursor to synthesize crystalline graphitic CDs.

**Figure 2:**
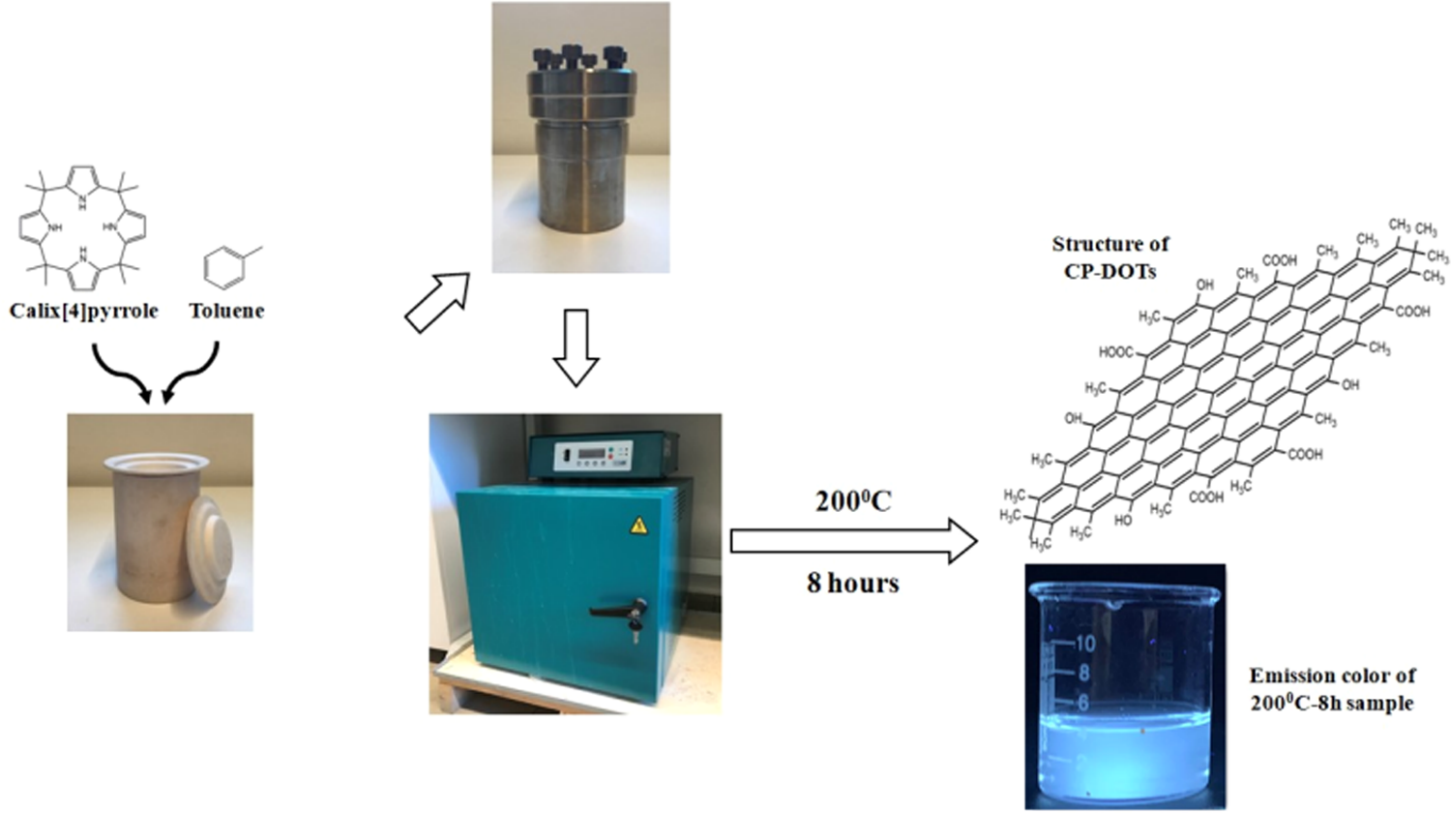
Schematic representation of the synthesis of CP-DOTs. The final product had a bright blue emission under 366 nm UV light.

In our study, CP-DOTs were prepared by a modified hydrothermal synthesis method using calix[4]pyrrole (CP) as the carbon precursor and toluene as the solvent (Figure 2). The optimum conditions for CP-DOT synthesis were determined as 200 °C for reaction temperature and 8 h for reaction time (Figure 2). For comparison with CP-DOTs, different carbon dots were synthesized under the same experimental conditions using either different carbon precursors or different solvents.

### Characterization of the Photophysical Properties of CDs

**Figure 3:**
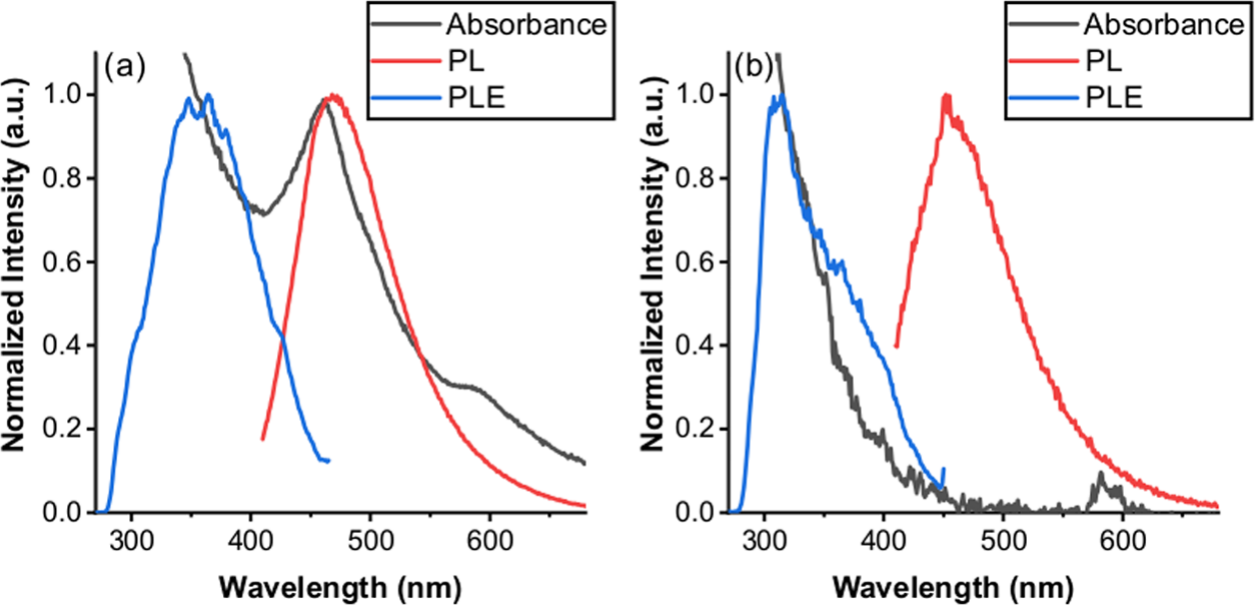
Emission—PL (red), absorption (black), and photoluminescence excitation—PLE (blue) spectra of (a) CP-DOTs and (b) T-DOTs. For the PL spectra, each sample was excited at 400 nm. In the absorption spectrum, the absorbance of each sample at 350 nm was adjusted to 0.1. The PLE spectrum of each sample was collected at the highest peak point of the emission spectrum. The PL and PLE spectra of each sample were normalized to 1 at the highest peak point. The absorption spectrum of each sample was normalized to 1 at an appropriate wavelength to have a better visual understanding.

The absorption spectrum of CP-DOTs had typical absorption bands observed for conventional carbon dots in the literature,^18−20,22,36^ with an extra band around 460 nm, which was attributed to the crystal band edge absorption (Figure 3a). The emission spectrum of CP-DOTs exhibited an excellent gaussian-shaped peak around 470 nm at excitation wavelength (λ_exc_) 400 nm (Figure 3a). The photoluminescence excitation (PLE) spectrum of the CP-DOTs (taken at an emission wavelength (λ_ems_) 470 nm) had a single peak around 360 nm with a smooth gaussian distribution (Figure 3a). Also, the photostability of CP-DOTs was measured by constantly illuminating the CP-DOT solution at λ_exc_ = 350 nm for 1 h. The emission of CP-DOTs stayed stable for 1-h illumination, which indicated that the CP-DOTs were very photostable (Figure S1). When the photophysical properties of the carbon dots synthesized using only toluene (T-DOTs) were compared with those of CP-DOTs, significant differences were observed (Figure 3b). T-DOTs had the typical absorption peaks of carbon dots, but did not have the crystal band edge absorption peak that the CP-DOTs had^18−20,22,36^ (Figure 3a,b). This clear difference was considered as a strong evidence for the formation of a smooth nanocrystalline structure for CP-DOTs.^18−20,22,36^ At λ_exc_ = 400 nm, the emission peak of T-DOTs was around 455 nm with a slightly distorted gaussian distribution. The full width at half-maximum (FWHM) was comparable for the emission peaks of both carbon dots: 95 nm for CP-DOTs and 105 nm for T-DOTs (Figure 3a,b). The PLE spectrum of T-DOTs (taken at λ_ems_ = 455 nm) had a broadened non-gaussian peak with a peak maximum at 310 nm (Figure 3b). All of these observations indicated that when CP was used as a carbon precursor in toluene, CDs with a very clear crystal band edge absorption peak and thus a smoother crystal structure was formed. It was also observed that CP-DOTs had an emission peak at a lower energy compared to T-DOTs. In addition, in the absence of CP, toluene was still able to induce the formation of carbon dots, but T-DOTs did not have as smooth a crystalline structure as the CP-DOTs.

**Figure 4:**
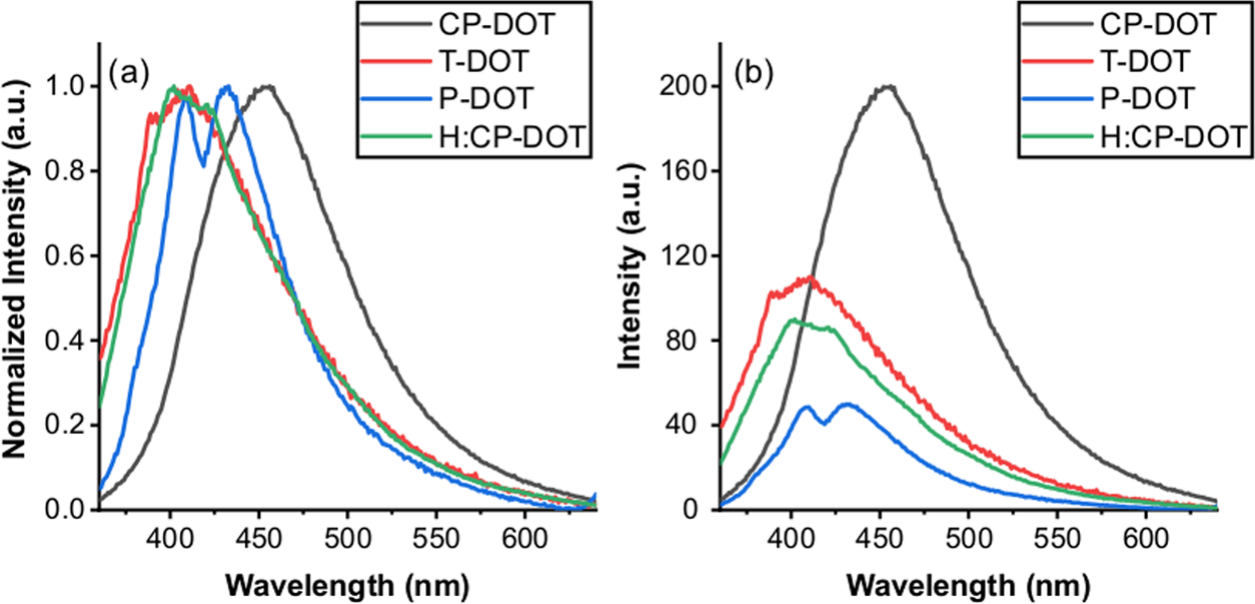
(a) Normalized and (b) actual emission spectra of CDs with different structures. Each sample was excited at 350 nm and the absorbance of each sample at 350 nm was 0.1. In the normalized emission spectrum, the highest point at each emission spectrum was normalized to 1.

When the emission peaks of T-DOTs and CP-DOTs with the same absorbance at 350 nm were compared at λ_exc_ 350 nm, it was observed that the emission peak of T-DOTs was a single non-gaussian peak at 405 nm with an intensity almost half of the intensity of the emission peak of CP-DOTs, which is a single Gaussian peak at 455 nm (Figure 4). As the carbon precursor was changed from CP to pyrrole (P-DOTs), carbon dots were obtained with lower quantum yields (Figure 4). Double-emission peaks at 410 nm and 432 nm were observed in the emission spectrum of P-DOTs, which showed that two dominant emission states arose when pyrrole was used as the carbon precursor. This characteristic behavior in the emission spectrum of P-DOTs may be due to two reasons: (1) formation of polydisperse carbon dots, and (2) formation of a high amount of defect states in the structure of the carbon dot.^37^ Moreover, the intensity of the double-emission peak of the P-DOTs was almost one quarter of the intensity of the emission peak of the CP-DOTs (Figure 4). Also, when the solvent was changed for the synthesis of CP-DOTs, the photophysical properties of CP-DOTs were significantly affected. As the toluene was replaced by n-hexane, the synthesized carbon dots (H:CP-DOTs) had a non-gaussian emission peak at 400 nm. Moreover, the intensity of the emission peak of H:CP-DOTs was almost half of the intensity of the emission peak of CP-DOTs. The QYs of CP-DOTs, T-DOTs, P-DOTs, and H:CP-DOTs were found to be 61%, 29%, 15%, and 25%, respectively. The difference between the QYs of CP-DOTs and H:CP-DOTs was expected because in the literature, it was observed that when amphiphilic CDs were synthesized in either toluene or hexane, CDs with smoother crystalline structure formed in toluene.^19^ Also, as was observed in the absorption spectrum of T-DOTs and CP-DOTs, T-DOTs had a less smooth crystal structure compared to CP-DOTs, which can be the reason for the lower QY of T-DOTs. In brief, the high quantum yield and perfect Gaussian-shaped emission peak of CP-DOTs pointed to the formation of monodisperse carbon dots with lesser defect states.

**Figure 5:**
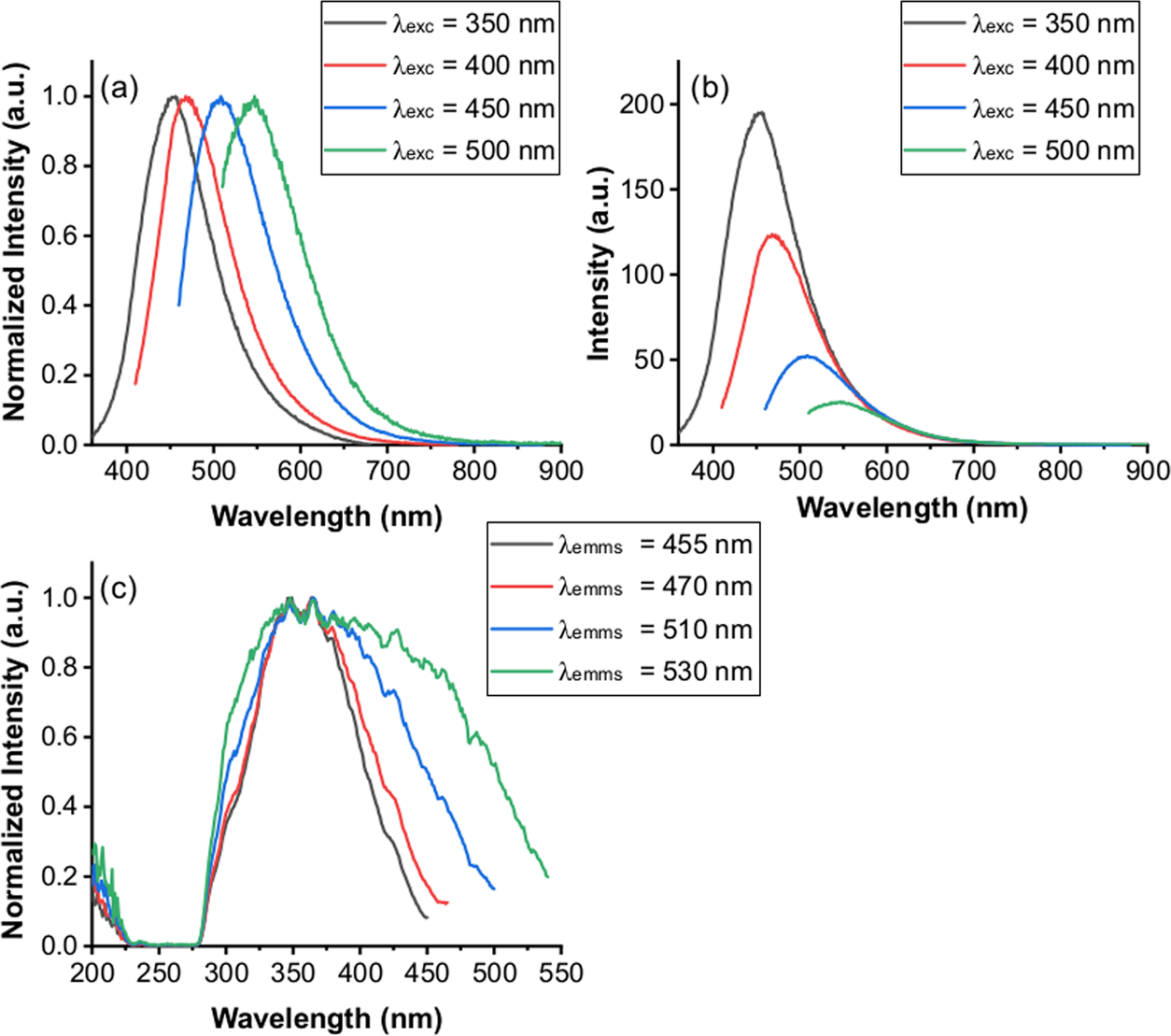
(a) Normalized emission, (b) actual emission, and (c) PLE spectra of CP-DOTs. In the normalized emission spectra, each spectrum was normalized to 1 at the highest peak point. In the PLE spectra, each spectrum was collected at the emission wavelength, with the highest peak point in the emission spectrum collected with different excitation wavelengths and normalized to 1 at the highest peak point.

The emission spectrum of CP-DOTs collected at different excitation wavelengths showed that CP-DOTs have excitation-dependent emission properties (Figure 5). An emission peak was observed at 455 nm when the CP-DOTs were excited at 350 nm. When the excitation wavelength shifted towards a lower-energy region, the emission peak maximum shifted towards the lower-energy region as well (Figure 5). The emission peak maxima were observed at 470 nm at λ_exc_ 400 nm, 510 nm at λ_exc_ 450 nm, and 530 nm at λ_exc_ 500 nm. The emission intensity of CP-DOTs was the highest when CP-DOTs were excited at 350 nm, and gradually decreased as the excitation wavelength shifted towards the lower-energy region (Figure 5). Also, it should be noted that CP-DOTs had a single, Gaussian peak in their PLE spectrum; however, the peak became broadened as the emission peak at which the PLE spectrum was collected shifted towards the lower-energy region. PLE spectra were collected at emission wavelengths where emission peak maxima were observed for different excitation wavelengths (Figure 4). The excitation-dependent emission properties of carbon dots can be observed due to the oxidation of the surface of the carbon dots.^38,39^ These results indicated that although there were no oxygen atoms in the molecular structure of CP, the surface of the CP-DOTs could be oxidized, and as a consequence, excitation-dependent emission properties were observed.

### Structural Characterization of CP-DOTs

In order to understand the crystal structure, size, bonding characterization, and surface characteristics of CP-DOTs, a series of measurements (e.g., TEM, XPS, and FTIR) were conducted on CP-DOTs.

**Figure 6:**
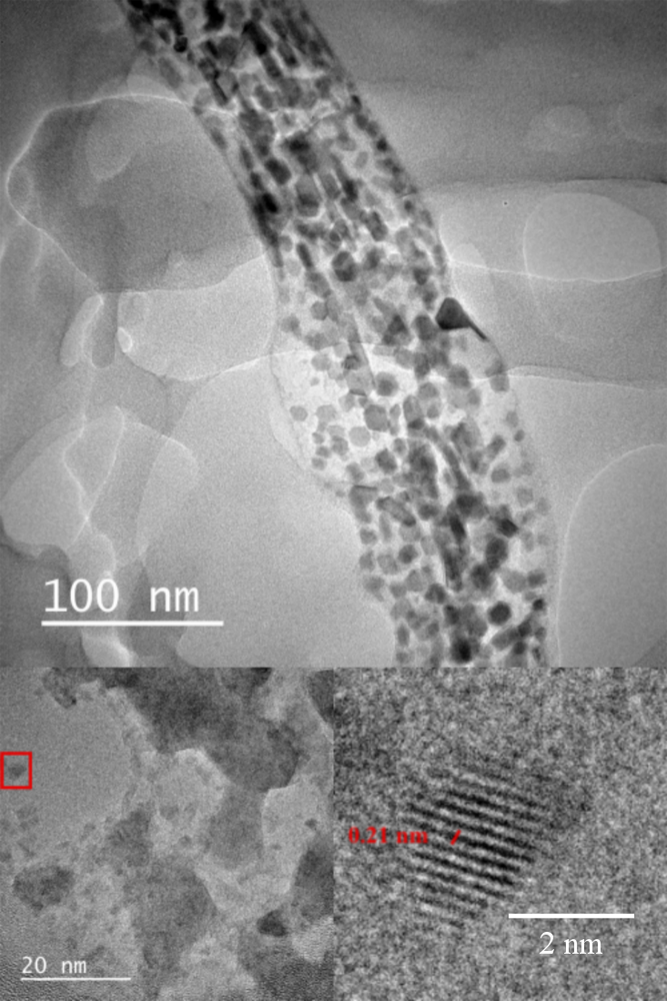
TEM (above) and HR-TEM (below) images of CP-DOTs.

The TEM images of CP-DOTs revealed that CP-DOTs had sizes in the range of 4–10 nm with a smooth lattice structure (Figure 6). In addition, HR-TEM results showed that CP-DOTs had a crystal structure with an interplanar distance of 0.21 nm, which corresponds to the (1100) lattice distance of graphene quantum dots.^40,41^ To completely understand the structure of CP-DOTs, FTIR and XPS spectra of the CP-DOTs were collected and compared with those of CP, the carbon precursor.

**Figure 7:**
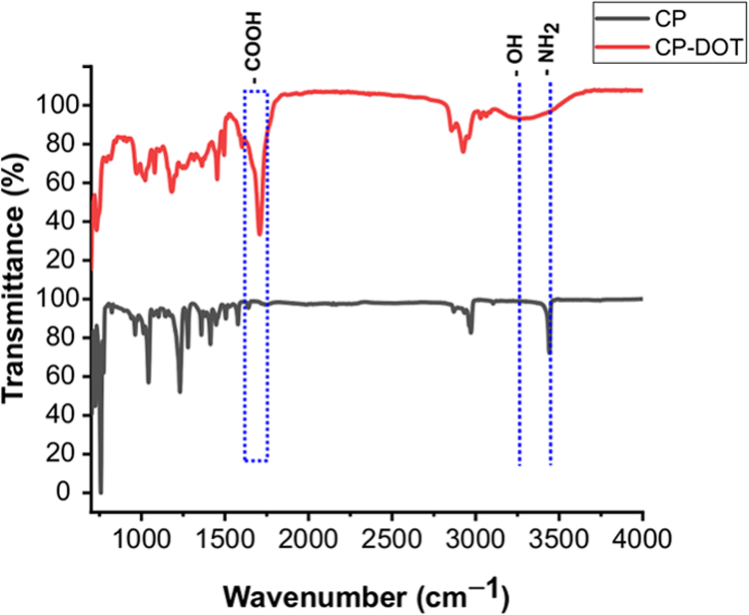
FTIR spectrum of CP (black) and CP-DOTs (red).

The FTIR spectrum of CP-DOTs had significant differences when compared to that of CP. The most pronounced difference was the appearance of the peak at 1700 cm^–1^ in the FTIR spectrum of CP-DOTs, while this peak was not observed in the FTIR spectrum of CP (Figure 7.). This peak was attributed to the presence of carboxylate groups (-COOH) on the surface of CP-DOTs and supported the excitation-dependent emission characteristics of CP-DOTs. The formation of carboxylic groups on the surface of carbon dots could be explained by the dissolution of oxygen in toluene at a high temperature and pressure. Hence, the dissolved oxygen in toluene caused the surface of the CP-DOTs to be oxidized, a phenomenon that was also reported in the literature.^18,19^ Additionally, the −NH band of CP at 3434 cm^–1^ completely disappeared in the FTIR spectrum of the CP-DOTs, indicating the nonparticipation of the nitrogen atoms present in the structure of CP. Furthermore, the existence of a broad–shallow band at 3100–3600 cm^–1^ was another evidence for the presence of −OH groups on the surface of CP-DOTs (Figure 7).

**Figure 8:**
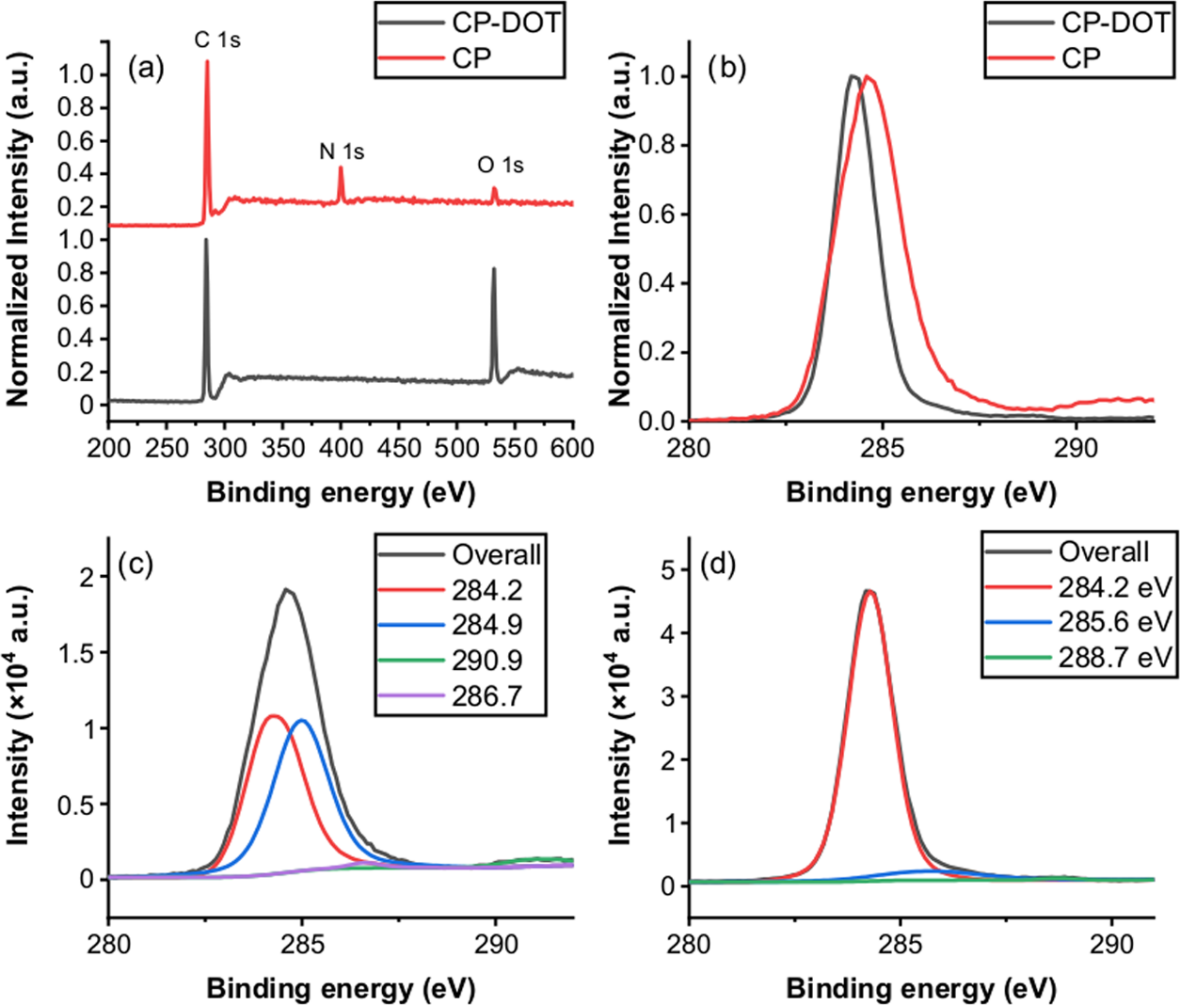
(a) XPS survey and (b) HR-XPS survey of C 1s of CP (red) and CP-DOTs (black). Each spectrum was normalized to 1 at the highest peak point. (c) Deconvoluted HR-XPS survey of C 1s of CP and (d) deconvoluted HR-XPS survey of C 1s of CP-DOTs.

Even more striking results were obtained when the XPS spectra of CP and CP-DOTs were compared. The most obvious difference observed was the complete disappearance of the nitrogen 1s peak in the XPS survey of the CP-DOTs (Figure 8). The atomic ratio of N:C in the XPS survey of CP was 1:7, which was perfectly coherent with the molecular structure of CP (C_28_H_36_N_4_). Also, a small fraction of oxygen (4%) was observed in the XPS spectrum of CP, which could be attributed to the adsorbed oxygen on the surface of the XPS experimental setup. On the other hand, the amount of oxygen significantly increased (28%) in the XPS survey of CP-DOTs, which could not be attributed only to the adsorbed oxygen and meant that oxygen was present in the structure of CP-DOTs (Figure 8). When the HR-XPS spectra of CP and CP-DOTs for the carbon 1s peak were compared, it was observed that the C 1s peak of CP-DOTs was narrower (Figure 8). When both spectra were deconvoluted, the C 1s peak of CP possessed 2 major peaks and one shallow peak at 284.2, 284.9, and 286.7 eV, which could be attributed to the sp^2^ hybridized C atoms, sp^3^ hybridized C atoms, and C-N bonding, respectively. However, the C 1s peak of CP-DOTs possessed one dominant peak at 284.2 eV and an emerging peak at 285.6 eV, which could be ascribed to the sp^2^ hybridized carbons and newly formed C=O bonding, respectively^18,19^ (Figure 8). All of these observations indicated that the nitrogen atom in the CP structure completely escaped during formation of CP-DOTs. Also, graphitic CDs with a dominant presence of sp^2^ hybridized carbons in their structure and carboxylate groups on the surface were formed when CP was used as the carbon precursor and toluene as the solvent.

### Effect of Solvents on the Photophysical Properties of CP-DOTs

**Figure 9:**
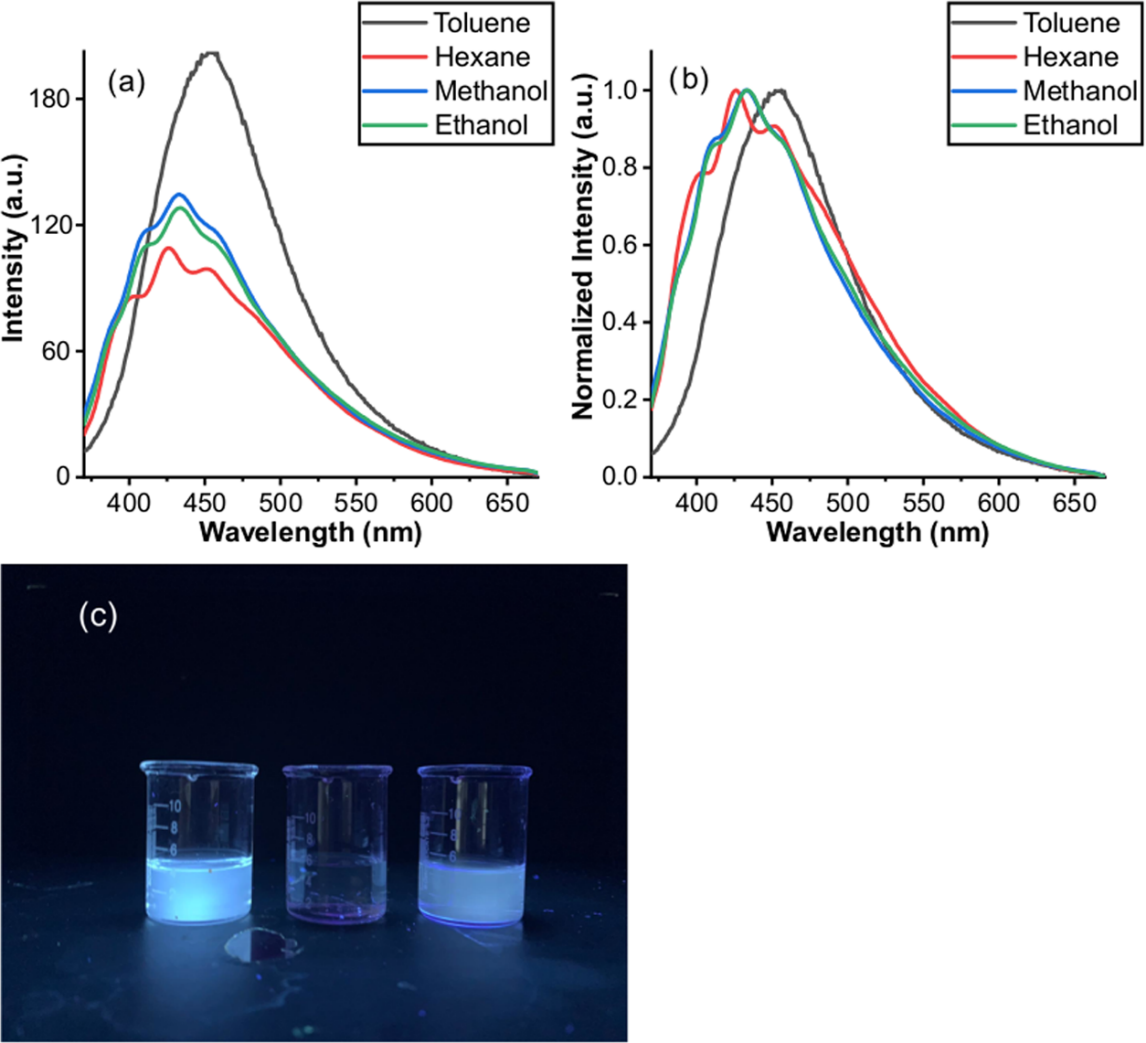
(a) Actual emission spectra, (b) normalized emission spectra, and (c) photoluminescence under UV light (right) of CP-DOTs in various solvents. In the normalized emission spectrum, each spectrum was normalized to 1 at the highest peak point. CP-DOTs were dispersed in toluene (left), in water (middle), and in methanol (right) to observe the photoluminescence under UV Light of CP-DOTs. Photograph courtesy of “Caner Ünlü.” Copyright 2022.

The presence of carboxylate groups on the surface of CP-DOTs is supposed to make CP-DOTs soluble in polar solvents. Thus, the solubility of CP-DOTs was also checked in several polar solvents. It was observed that while CP-DOTs were not soluble in pure water, they were soluble in ethanol and methanol (Figure 9). This characteristic feature can also be observed in fatty acids and fatty acid salts, such as myristic acid, stearic acid, sodium myristate, etc.^32^ Although CP-DOTs were soluble in methanol and ethanol, the quantum yield of CP-DOTs dropped by at least 40% in these solvents. It should be noted that the quantum yield of CP-DOTs decreased even more (by 50%) when CP-DOTs were dissolved in hexane. Additionally, the emission peak of CP-DOTs in methanol, ethanol, and hexane was blue-shifted, along with distortions in the shape of the peaks (Figure 9). All of these observations pointed out that CP-DOTs were most stable in toluene and soluble in polar solvents, which means CP-DOTs should be considered as amphiphilic rather than hydrophobic. Also, the emission characteristics of CP-DOTs showed variations in different solvent systems due to the presence of carboxylic groups on the surface of CP-DOTs and the difference in the interaction between the solvents and the surface of CP-DOTs.

### CP-DOTs in Organic Solar Cells: Optical, Structural, and Morphological Characterization of the Photoactive Layer

**Figure 10:**
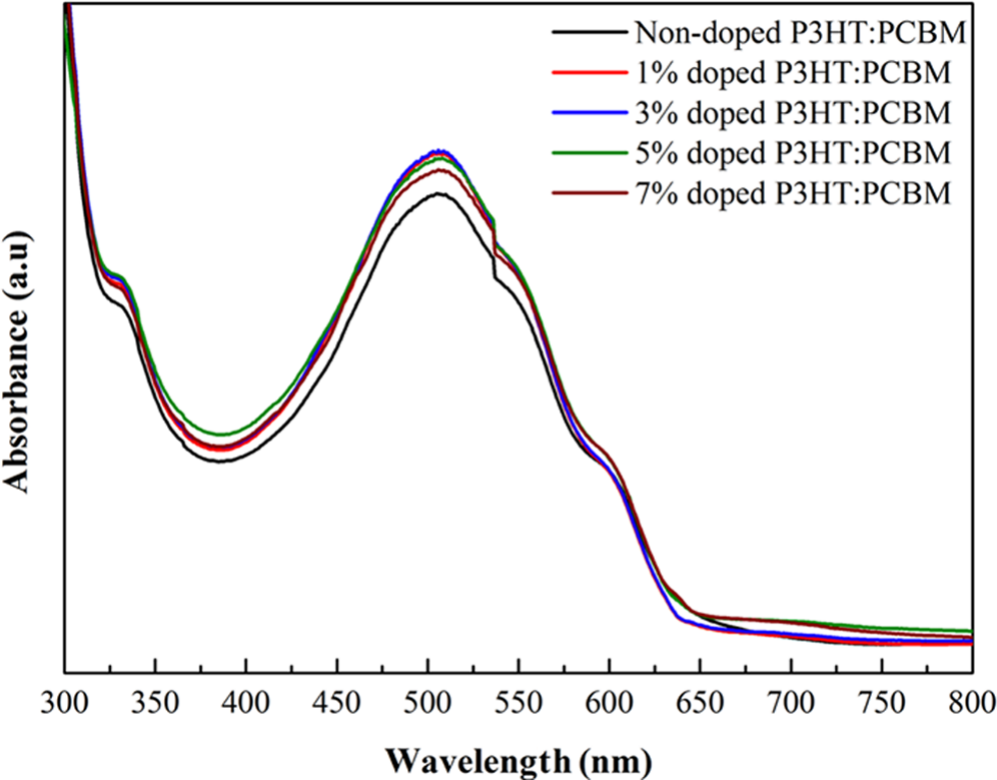
UV–vis absorption spectra of the nondoped and doped films of P3HT:PCBM.

In order to understand the effect of addition of CP-DOTs into the P3HT:PCBM film, UV–vis, XRD, and AFM measurements were carried out. The UV–vis spectrum was recorded to obtain the optical absorption spectra of nondoped and doped P3HT:PCBM films in the range from 300 to 800 nm (Figure 10). In the absorption spectra of nondoped and doped P3HT:PCBM films, similar behaviors were observed. In each spectrum, the absorption shoulders were observed at 330, 500, and 600 nm. The absorption peak at 330 nm was attributed to PCBM, whereas the peaks at 500 and 600 nm were attributed to P3HT.^42^ Additionally, the peak at 500 nm hinted the generation of one exciton and two photons.^42^ The results of UV–vis spectra also showed that the intensity of absorption increased slightly as the presence of CP-DOTs increased. Even when there were slight differences between the absorption intensities, the presence of CP-DOTs dramatically affected the optical properties of OSCs.

**Figure 11:**
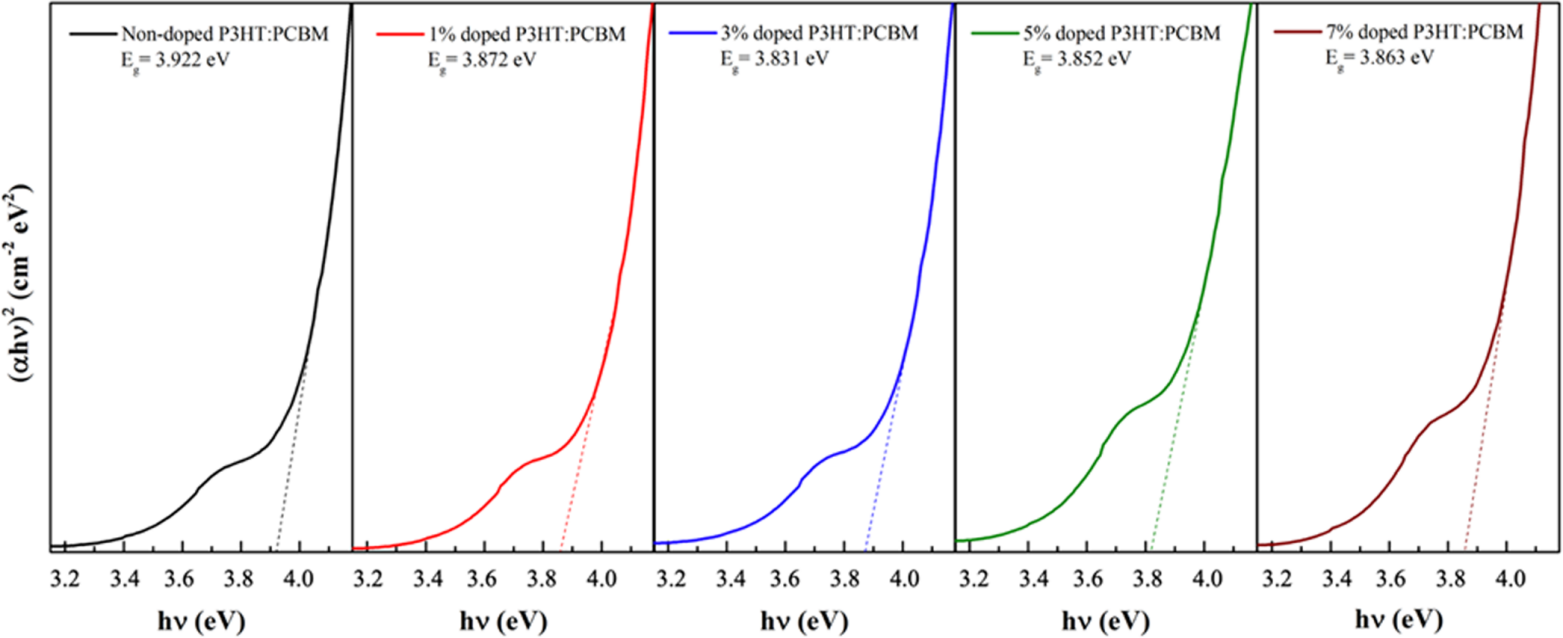
Optical band gap of the nondoped and doped films of P3HT: PCBM extracted from Tauc’s lot.

The optical band gap (*E*_g_) of nondoped and doped P3HT:PCBM films was calculated from the Tauc plot (Figure 11)^43^ by following eq 1
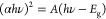
1where α *is* the absorption coefficient*, h*ν is the photon energy, and *A* is an independent constant. The P3HT:PCBM films used in this study had an average thickness of 200 nm. As summarized in Table 1, the *E*_g_ values of the P3HT:PCBM films decreased from 3.922 eV (for the nondoped P3HT:PCBM film) to 3.872, 3.831, 3.852, and 3.863 eV after the addition of a stock solution of CP-DOTs of 1, 3, 5, and 7 vol %, respectively. It can be clearly concluded that the *E*_g_ values decreased as the amount of CP-DOTs increased. The absorption range slightly increases in the visible region, which corresponds to a decrease in the band gap.^44^ Nevertheless, a higher doping concentration value would lead to increase in the *E*_g_ value. This could be explained by the Burstein-Moss (BM) effect, which is related to the free electron density.^45−47^

**Table 1:** Optical Band Gap, Crystal Size, and Roughness Values with Nondoped and CP-DOT-Doped CQD P3HT:PCBM Films

	*E*_g_ (eV)	crystallite size (nm)	roughness (nm)
nondoped P3HT:PCBM	3.922	18.32	1.307
1% doped P3HT:PCBM	3.872	22.18	1.167
3% doped P3HT:PCBM	3.831	23.90	1.096
5% doped P3HT:PCBM	3.852	21.92	1.113
7% doped P3HT:PCBM	3.863	20.90	1.201

**Figure 12:**
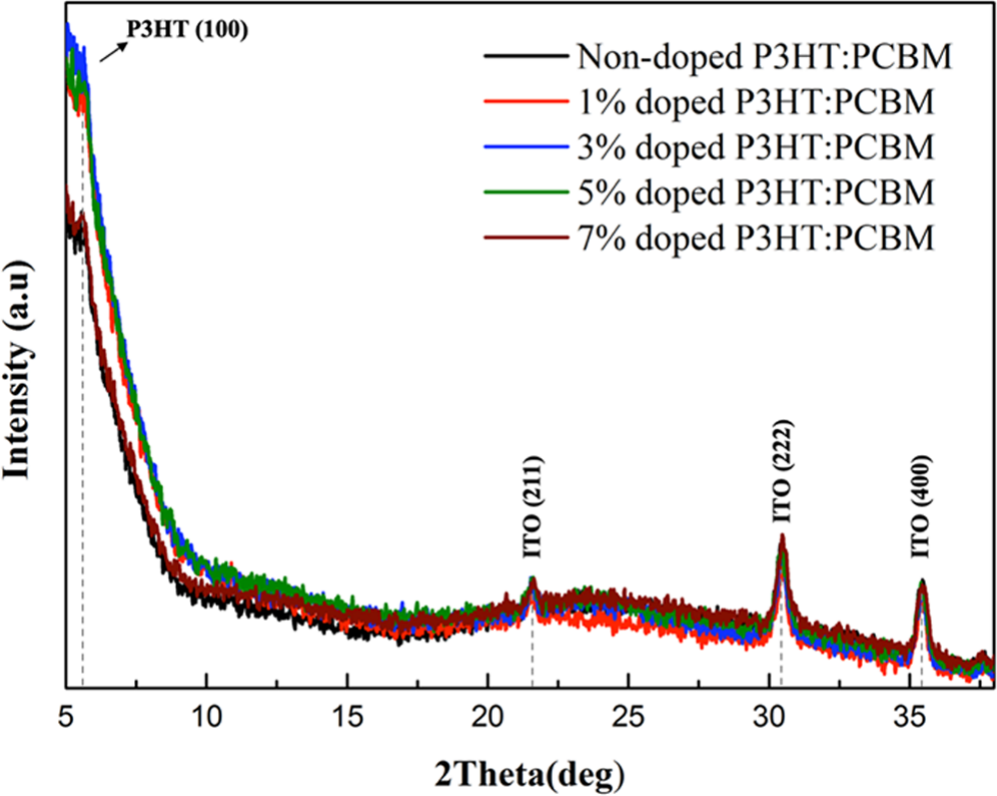
XRD spectra of the nondoped and doped films of P3HT:PCBM.

The crystallinity of P3HT is directly related to the charge carrier mobility and therefore could play a critical role for OSCs performance.^48^ Therefore, XRD spectra of the nondoped and doped films of P3HT: PCBM were used to analyze the improvement of crystallinity of P3HT (Figure 12). The XRD patterns clearly showed that peaks of P3HT exhibited at around a 2θ value of 5.6° corresponding to the (100) reflection peak. The XRD peaks identifying the polycrystalline structure of ITO were attributed to (211), (222), and (400) planes.^49^ The intensity of the XRD peaks in the CP-DOT-doped films slightly increased, which showed that the crystallinity of P3HT was increased with the doping vol % of CP-DOTs. The mean size of P3HT crystallinity was measured by Scherrer equation,^50^ and the results indicated that the P3HT crystallinity sizes increased from 18.32 nm for the nondoped to 22.18, 23.90, 21.92, and 20.90 nm for the 1, 3, 5, and 7 vol % of CP-DOT-doped P3HT: PCBM blend, respectively (Table 1). The biggest crystallinity size was observed to be 23.90 nm from 3 vol % of CP-DOTs. As the correlation between carrier mobility and crystallinity was considered, the carrier mobility should increase with better crystallinity for P3HT-based films.^51^ According to these results, using CP-DOTs as an additive increased the crystallinity of P3HT, and as a consequence, CP-DOTs can be considered as an excellent additive candidate to improve OSCs’ photovoltaic performance due to the increments in the charge carrier mobility of the P3HT:PCBM layer.^52,53^

**Figure 13:**
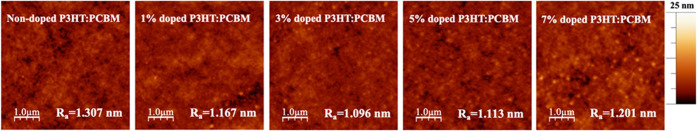
AFM images of the nondoped and doped films of P3HT:PCBM.

The morphology of P3HT:PCBM films has an influence on the photovoltaic properties of fabricated OSCs.^54^ Hence, the AFM technique was used to investigate the morphology of P3HT:PCBM films. The average roughness (*R*_a_) of P3HT:PCBM films was measured using WSxM 5.0 software.^55^ The *R*_a_ values of nondoped P3HT:PCBM films was measured as 1.307 nm and was reduced for 3 vol % CP-DOT-doped P3HT:PCBM films to 1.096 nm, which was the lowest observed R_a_ value after the addition of CP-DOTs (Figure 13). Since the charge transport properties of the photoactive layer extremely depend on its morphology, the improvement of the photoactive layer with different concentrations of CP-DOTs has an importance for the enhancement of OSCs’ photovoltaic parameters.^56^

### CP-DOTs in Organic Solar Cells: Characterization of OSCs

**Figure 14:**
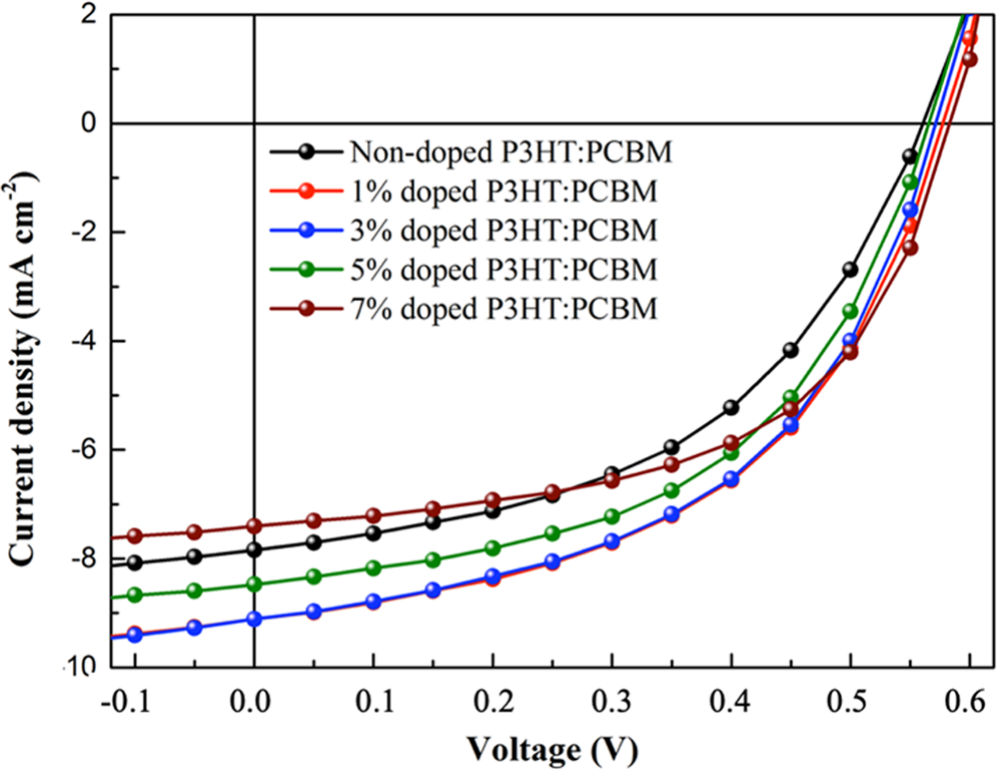
Current density–voltage (*J*–*V*) curves of all of the OSCs with different volumes of CP-DOTs under an illumination of AM 1.5 G, 80 mW cm^–2^.

OSCs were prepared based on the following structure: Glass/ITO/PEDOT:PSS (40 nm)/P3HT:PCBM (200 nm)/LiF:Al (2 nm:80 nm). The nondoped and doped P3HT:PCBM-based OSCs were fabricated to investigate the effect of CP-DOT addition on the photovoltaic parameters by measuring the current density–voltage (*J*–*V*) curves of the OSCs (Figure 14). Table 2 summarizes the photovoltaic parameters obtained from the *J*–*V* curves.

**Table 2:** Device Figure-of-Merit Parameters of the Organic Solar Cells with Nondoped and CP-DOT-Doped Films under an Illumination of AM 1.5, 80 mW/cm^2^

	FF (%)	*V*_oc_ (V)	*J*_sc_ (mA cm^–2^)	PCE (%)	*R*_s_ (Ω cm^–2^)	*R*_sh_ (Ω cm^–2^)
nondoped P3HT:PCBM	48.51	0.561	7.842	2.66	24.05	216.64
1% doped P3HT:PCBM	48.03	0.578	9.112	3.16	22.21	283.85
3% doped P3HT:PCBM	52.15	0.571	9.113	3.39	15.56	309.59
5% doped P3HT:PCBM	51.98	0.566	8.477	3.12	20.26	292.31
7% doped P3HT:PCBM	53.26	0.582	7.405	2.87	23.56	272.40

The photovoltaic parameters of OSCs were enhanced after addition of CP-DOTs. The OSCs prepared with the nondoped P3HT:PCBM blend solution presented a fill factor (FF) of 48.51%, an open-circuit voltage (*V*_oc_) of 0.561 V, a short circuit current density (*J*_sc_) of 7.841 mA cm^–2^, and a lower PCE value of 2.66%. The best device parameters were obtained to be a PCE of 3.39%, which was calculated from an FF of 51.15%, a *V*_oc_ of 0.571 V, and a *J*_sc_ of 9.113 mA cm^–2^ after 3 vol % of CP-DOT addition. FF and *J*_sc_ are important parameters for the enhancement of PCE.^57^ In addition, the trap density, carrier mobility, diffusion length, and carrier lifetime are also affected by these parameters.^58^ In comparison to the nondoped OSCs, *V*_oc_ and *J*_sc_ values increased after the addition of CP-DOTs. On the other hand, a higher doping concentration can cause a decrease in *J*_sc_ value as it can lead to a narrow depletion region, which is a drawback for carrier accumulation^59^ and can explain the lowe *J*_sc_ value (7.405 mA) for 7 vol % of CP-DOT addition. As a result, the PCE value of 7 vol % of CP-DOT addition decreased to 2.87%. The increased J_sc_ value of the prepared OSCs with 1 and 3 vol % of CP-DOT addition could be related to the synergistic effects of CP-DOT addition to the morphology of the prepared photoactive layer. This result is also due to the increase in photocarriers because of the improved light efficiency of the OSCs.^60^ However, the *J*_sc_ values started to decrease after 5 vol % of CP-DOT addition. On the other hand, *V*_oc_ values strongly depend on the crystallinity, trap density, and morphology of the photoactive layer.^61,62^ It can be said that these results are attributed to the structural and morphological properties of the photoactive layer, which are consistent with the optical absorption data, XRD data, and AFM results.^63^ The value of FF increased from 48.51 to 52.15, 51.98, and 53.26% with the addition of 3, 5, and 7 vol % CP-DOTs, respectively, while it decreased to 48.51% with the addition of 1 vol % CP-DOTs. With an exception of 1 vol % CP-DOTs, the value of FF increased with the rising concentration of CP-DOTs. These results indicate that the charge generation, ohmic contact, and extraction efficiency of the photoactive layer enhanced with the addition of CP-DOTs.^64^

To obtain more details regarding the charge transport properties, serial resistance (*R*_s_) and shunt resistance (*R*_sh_) were measured from the *J–V* curves. The values of *R*_s_ and *R*_sh_ are also given in Table 2. As is well known, *R*_s_ and *R*_sh_ play a significant role in increasing the FF and *J*_sc_ values, which are two important parameters that improve the photovoltaic performance. A decrease in *R*_s_ values but increase in *R*_sh_ values is expected after the addition of CP-DOTs.^65^ The lowest 15.56 Ω cm^–2^ of *R*_s_ and the highest 309.59 Ω cm^–2^ of *R*_sh_ were acquired from 3 vol % of CP-DOT addition into the P3HT:PCBM-based OSCs. It can be said that the enhancement of FF values with an exception (as mentioned above) is in good match with these results as *R*_sh_ is related to the charge transport properties and leakage current.^53^ To sum up, in the best case, the PCE of OSCs increased by 50% upon addition of 3% vol of CP-DOT dopant solution to the active layer, which shows that CP-DOTs showed a synergistic effect and enhanced photovoltaic parameters of traditional organic solar cells. After these values were compared with the other OSCs studied in the literature (Table S2), it was concluded that addition of CP-DOTs to the active layer in OSCs is one of the most effective ways to improve the photovoltaic parameters of OSCs.

## Conclusions

Water-insoluble, slightly amphiphilic CDs with high quantum yield and smooth crystal structure were synthesized using calix[4]pyrrole as the carbon precursor and toluene as the solvent. Carbon dots had a size of around 4–10 nm with graphitic structure. The quantum yield of carbon dots was calculated to be 61%. Amphiphilic quantum dots were soluble in toluene, hexane, ethanol, and methanol, and were brightest in toluene. CP-DOTs possessed excitation-dependent emission properties because of the existence of carboxylic groups on their surface. Furthermore, CP-DOTs as an additive were utilized to improve the OSCs’ photovoltaic parameters. The P3HT:PCBM layer with addition of different vol % (nondoped, 1, 3, 5, and 7%) of CP-DOTs was used to fabricate the OSCs. The optical absorption, crystallinity, and morphological properties of the photoactive layer were improved after addition of carbon dots in the P3HT:PCBM layer. In addition, all device parameters were enhanced after addition of CP-DOTs in the photoactive layer. According to the nondoped OSCs, FF increased from 48.51 to 52.15% and J_sc_ increased from 7.842 to 9.113 mA cm^–2^, which led to an increase in PCE from 2.66 to 3.39% by adding 3 vol % carbon dots. The adaptation of CP-DOTs to solar cell systems could contribute to commercialization and scale-up of solar cell technology with increase of OSC device parameters. Finally, our results showed that calix[4]pyrroles can be considered as powerful carbon precursors to synthesize graphitic–amphiphilic CDs, and in future studies, calix[4]pyrrole derivatives with different functional groups can be used to synthesize heteroatom-doped amphiphilic CDs. Furthermore, considering the photovoltaic parameters from the OSC results we have obtained, CP-DOTs could be an excellent candidate for improving optoelectronic devices.

## Supporting Information Available

Change in intensity of CP-DOTs upon continuous illumination with λ_exc_ = 350 nm, Device Figure-of-Merit Parameters of the Organic Solar Cells in literature (PDF)

## Notes

C.Ü. has received research grants from ITU-BAP (Istanbul Technical University—Scientific Research Projects Unit). The authors declare that they have no conflict of interest.
