# Supplementary material for: Development of Highly Luminescent Water-Insoluble Carbon Dots by Using Calix[4]pyrrole as the Carbon Precursor and Their Potential Application in Organic Solar Cells
Source: ACS Omega. 2022 May 24;7(22):18840–51. doi: 10.1021/acsomega.2c01795 (PMC9178622; doi:10.1021/acsomega.2c01795)
Supplement: Supplementary file 1 — ao2c01795_si_001.pdf [file ao2c01795_si_001.pdf]

SUPPORTING INFORMATION

Development of Highly Luminescent Water Insoluble Carbon Dots by using Calix[4]pyrrole as Carbon Precursor and Their Potential Application in Organic Solar Cells

Yağız Coşkun[a,1], Fatma Yelda Ünlü[b,1], Tuğbahan Yılmaz[c,1,*], Yurdanur Türker[d], Abdullah Aydogan[b], Mahmut Kuş[e], Caner Ünlü[a,b,f,*]

a. Istanbul Technical University, Department of Nanoscience and Nanoengineering, Maslak, 34469 Istanbul, Turkey

b. Istanbul Technical University, Faculty of Science and Letters, Department of Chemistry, 34469, Maslak, Istanbul, Turkey

c. Konya Technical University, Vocational School of Technical Sciences, Department of Electricity and Energy, 42150, Selcuklu, Konya, Turkey

d. Sabanci University Nanotechnology Research & Application Center (SUNUM), Sabanci University, Istanbul, Turkey

e. Konya Technical University, Department of Chemical Engineering, 42075, Konya, Turkey

f. Istanbul Technical University Nanotechnology Research and Application Center (ITUNano), Istanbul, Turkey

1 Authors contributed equally

* Corresponding authors

E-mail address of corresponding authors: canerunlu@itu.edu.tr; tgbhn.ylmz@gmail.com

E-mail address of all authors by author order:

i.yagizcoskun@gmail.com, unlu19@itu.edu.tr, tgbhn.ylmz@gmail.com, yurdanur.turker@sabanciuniv.edu, aydoganab@itu.edu.tr, mahmutkus1@gmail.com, canerunlu@itu.edu.tr

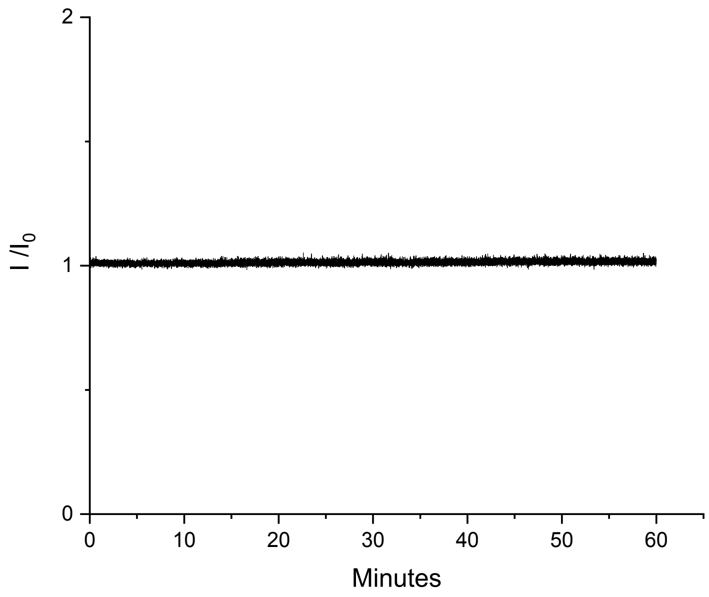

Figure S1. Change in intensity of CP-DOTs upon continuous illumination with λ_{exc}= 350 nm. I represents the emission intensity at 455 nm at corresponding time and I_0 represents emission intensity at 455 nm at time: 0 minute.

Table S1. Device Figure-of-Merit Parameters of the Organic Solar Cells in literature

Structure of OSCs	Content (vol. or wt. %)	FF	V_{oc} (mA)	J_{sc} (mA cm^{-2})	PCE (%)	Δ PCE with respect to non doped (%)	Ref.
ITO/PEDOT:PSS/P3HT:PCBM:B-CQDs/LiF-Al	w/o B-CQDs	0.32	0.581	7.603	1.78	0	(1)
	1 vol.% B-CQDs	0.47	0.563	7.901	2.66	49.44	
	3 vol.% B-CQDs	0.41	0.582	7.354	2.14	20.22	
	5 vol.% B-CQDs	0.38	0.569	7.839	2.69	51.12	
FTO/TiO_2/P3HT:PCBM:B-CQDs/MoO_3-Ag	Non-doped	0.31	0.515	8.437	1.72	0	(2)
	1 vol.% of B-QCDs	0.33	0.484	8.034	1.62	-5.81	
	3 vol.% of B-QCDs	0.39	0.546	8.606	2.33	35.46	
	5 vol.% of B-QCDs	0.36	0.538	9.264	2.25	30.81	
ITO/PEDOT:PSS/P3HT:PCBM:GNP/Ca-Al	0 wt.% GNP	0.38	1.19	5.61	2.54	0	(3)
	1 wt.% GNP	0.42	1.22	5.79	2.98	17.32	
	3 wt.% GNP	0.47	1.24	6.18	3.61	42.12	
	5 wt.% GNP	0.33	1.14	5.41	2.05	-19.29	
ITO/PEDOT:PSS/P3HT:PCBM:CdS/LiF-Al	0 wt. CdS	0.54	0.63	8.5	2.95	0	(4)
	0.2 wt. CdS	0.60	0.65	9.2	3.66	24.07	
	0.4 wt. CdS	0.63	0.64	10.8	4.41	49.49	
	0.6 wt. CdS	0.41	0.48	6.2	1.24	-57.96	
ITO/PEDOT:PSS/P3HT:PCBM:RGO/Al<br>ITO/PEDOT:PSS/P3HT:PCBM:N-RGO/Al	0 wt.%	0.51	0.58	10.54	3.12	0	(5)
	0.1 wt.% of RGO	0.40	0.53	12.69	2.71	-13.14	
	0.5 wt.% of RGO	0.40	0.54	7.37	1.61	-48.39	
	1 wt.% of RGO	0.38	0.45	6.57	1.06	-66.02	
	0.1 wt.% of N-RGO	0.50	0.56	12.96	3.65	16.98	
	0.5 wt.% of N-RGO	0.50	0.58	14.86	4.39	40.70	
	1 wt.% of N-RGO	0.46	0.58	14.86	4.06	30.12	
ITO/PEDOT:PSS/ P3HT:PTM21-GOS:PCBM /LiF:Al<br>ITO/PEDOT:PSS/ P3HT:PTM21-CNT:PCBM /LiF:Al	0 wt.%	0.28	0.52	7.18	1.05	0	(6)
	0.1 wt.% of PTM21-CNT	0.30	0.55	8.16	1.36	29.52	
	0.3 wt.% of PTM21-CNT	0.31	0.54	9.27	1.55	47.61	
	0.5 wt.% of PTM21-CNT	0.30	0.52	8.34	1.31	24.76	
	0.1 wt.% of PTM21-GOS	0.29	0.59	8.11	1.43	36.19	
	0.3 wt.% of PTM21-GOS	0.30	0.58	9.68	1.70	61.90	
	0.5 wt.% of PTM21-GOS	0.28	0.58	8.63	1.44	37.14	

REFERENCES:

(1) Kırbıyık Kurukavak, Ç.; Yılmaz, T.; Çetin, Ş.; Alqadasi, M. M.; Al-Khawlany, K. M.; Kuş, M. Synthesis of Boron-Doped CQDs and Its Use as an Additive in P3HT:PCBM Layer for Efficiency Improvement of Organic Solar Cell. *Microelectron. Eng.* **2021**, *235*. https://doi.org/10.1016/j.mee.2020.111465.

(2) Yilmaz, T. The Hydrothermal Synthesis of Blue-Emitting Boron-Doped CQDs and Its Application for Improving the Photovoltaic Parameters of Organic Solar Cell. *Turkish J. Chem.* **2021**, *45* (6). https://doi.org/10.3906/kim-2104-14.

(3) Aïssa, B.; Nedil, M.; Kroeger, J.; Ali, A.; Isaifan, R. J.; Essehli, R.; Mahmoud, K. A. Graphene Nanoplatelet Doping of P3HT:PCBM Photoactive Layer of Bulk Heterojunction Organic Solar Cells for Enhancing Performance. *Nanotechnology* **2018**, *29* (10). https://doi.org/10.1088/1361-6528/aaa62d.

(4) Imran, M.; Ikram, M.; Shahzadi, A.; Dilpazir, S.; Khan, H.; Shahzadi, I.; Yousaf, S. A.; Ali, S.; Geng, J.; Huang, Y. High-Performance Solution-Based CdS-Conjugated Hybrid Polymer Solar Cells. *RSC Adv.* **2018**, *8* (32). https://doi.org/10.1039/c8ra01813h.

(5) Jun, G. H.; Jin, S. H.; Lee, B.; Kim, B. H.; Chae, W. S.; Hong, S. H.; Jeon, S. Enhanced Conduction and Charge-Selectivity by N-Doped Graphene Flakes in the Active Layer of Bulk-Heterojunction Organic Solar Cells. *Energy Environ. Sci.* **2013**, *6* (10). https://doi.org/10.1039/c3ee40963e.

(6) Lee, R. H.; Huang, J. L.; Chi, C. H. Conjugated Polymer-Functionalized Graphite Oxide Sheets Thin Films for Enhanced Photovoltaic Properties of Polymer Solar Cells. *J. Polym. Sci. Part B Polym. Phys.* **2013**, *51* (2). https://doi.org/10.1002/polb.23180.
